# The effect of sweat sample storage condition on sweat content

**DOI:** 10.1080/23328940.2020.1867294

**Published:** 2021-01-15

**Authors:** Lisa Klous, Mireille Folkerts, Hein Daanen, Nicola Gerrett

**Affiliations:** Department of Human Movement Sciences, Faculty of Behavioural and Movement Sciences, Vrije Universiteit Amsterdam, Amsterdam Movement Sciences, Amsterdam, The Netherlands

**Keywords:** Duration, electrolytes, metabolites, sweat composition, sweat storage, temperature

## Abstract

Due to time and logistical constraints sweat samples cannot always be analyzed immediately. The purpose of this study was to investigate the effect of storage temperature and duration on sweat electrolyte and metabolite concentrations. Twelve participants cycled for 60 min at 40 W.m^−2^ in 33°C and 65% RH. Using the absorbent patch technique, six sweat samples were collected from the posterior torso. Sweat from the six samples was mixed, divided again over six samples and placed in sealed vials. Sweat sodium, chloride, potassium, ammonia, lactate and urea concentrations in one sample were determined immediately. Two samples were stored at room temperature (~25°C, 42% RH) for 7 and 28 days respectively. The remaining samples were frozen at −20°C for 1 h, 7 or 28 days respectively before analysis. Sweat sodium, chloride, potassium and urea concentrations were not affected by storage temperature and duration. Sweat lactate decreased (−1.8 ± 1.8 mmol.L^−1^, P = 0.007) and ammonia concentrations increased (5.1 ± 3.9 mmol.L^−1^, P = 0.017) after storage for 28 days at 25°C only. The storage temperature and duration did not affect sodium, chloride, potassium and urea concentrations. However, sweat samples should not be stored for longer than 7 days at 25°C to obtain reliable sweat lactate and ammonia concentrations. When samples are frozen at −20°C, the storage duration could be extended to 28 days for these components.

## Introduction

The collection of sweat samples and the subsequent determination of sweat composition is becoming common practice in sports science. Sweat electrolytes, such as sodium, chloride, and potassium, are important for maintaining fluid balance [1] and play a role in the evaporative heat loss ability from the human body [2]. In thermally challenging environments, sweating athletes could lose excessive amounts of fluid and electrolytes, which could impair performance [3,4]. To quantify these electrolyte losses and select the appropriate personalized replacement strategy, researchers and practitioners conduct sweat testing. Previous research reported large intra- and interindividual variation in sweat composition [1,5], which could be caused by fitness level, acclimation status, the environment, and diet. Other sources of variation may be associated with the different methods of sweat collection and analysis [1].

For whole-body sweat analysis, the most accurate method appears to be a whole-body washdown. This method accounts for all sweat lost from the body and it does not interfere with the process of sweating, allowing for the total loss of fluid and electrolytes to be quantified. However, this technique is restricted to the laboratory as all sweat should be collected and contamination with clothing should be prevented. For field-based measurements, the local absorbent patch technique [6] is typically used to collect sweat for chemical analysis. Baker and colleagues [7] have shown that whole-body sweat sodium loss can be predicted from the local absorbent patch technique with an acceptable level of accuracy.

Due to time and logistical constraints, sweat samples cannot always be analyzed immediately. Whilst several studies have described the best techniques for sweat collection and subsequent chemical analysis [8–10], the best practices for sweat sample storage are yet to be quantified. It has been suggested that the use of inconsistent storage conditions (i.e., temperature and duration) can lead to substantial errors [11–14]. Dziedzic et al. [12] reported a ≤ 14% increase in sweat sodium concentration when analyzed immediately after sampling compared to after refrigeration at 7°C for 7 days. Another study reported a 3–19% increase in simulated sweat chloride concentration when stored at 28°C for five days, compared to 21–66% when being stored at 21–23°C [13]. It could be that the increased concentrations are caused by evaporation (i.e., water loss) from the samples. Refrigerated sealed samples showed the least evaporation (2% increase in concentration after 5 days), whilst the most evaporation occurred in unsealed samples at room temperature (32% increase in concentration after 5 days) [13]. Therefore, sweat samples should be sealed or at least it should be reported whether sealing was adhered to [9]. More recently, Baker et al. [11] concluded that sodium, chloride and potassium concentrations decreased significantly after 7 days of storage at a range of temperatures (−20°C, 8°C, 23°C, and an alternation of 8°C and 23°C to simulate sample transport from the field to the laboratory). Lastly, guidelines for the diagnosis of cystic fibrosis through chloride testing in sweat recommend sample storage for a maximum of three days at 4°C in the event a sample cannot be analyzed immediately [15,16]. In brief, the results of the limited studies that are published on the best practices for sweat sample storage conditions are unequivocal.

The number of studies developing a wearable sweat sensor is rapidly increasing [17–21]. These devices typically aim to measure metabolites in sweat, because they are thought to relate to a state of fatigue, hydration and/or muscle cramps. The wearable sensors analyze the sweat sample immediately and therefore storage is not an issue. However, the development of such sensors is advancing far quicker than scientific knowledge on the physiological mechanisms determining the concentrations of metabolites in sweat. To improve our understanding of eccrine sweat composition, the number of studies measuring metabolites in sweat, such as lactate [22–27], ammonia [23,24] and urea [23,28], in experiments is increasing. Lactate, ammonia, and urea potentially relate to the eccrine sweat glands metabolism, but do not appear to (strongly) relate to their blood counterparts, implying absence of a relation with the muscle metabolism [1,5]. To implement the best practices for sample storage of metabolites in such experiments, the effect of storage condition (i.e., temperature and duration) on sweat metabolite concentrations has to be quantified. As earlier mentioned, there is high variability in sweat composition and large differences between sampling methods and storage practices may have contributed to this variability between labs. A review by Baker [9] highlighted that more research is needed to determine best practices for sample storage. We feel that our paper has an important contribution to the emerging field of sweat analytics.

The present study investigated the effect of sweat sample storage temperature and duration (25°C for 7, 28 days or −20°C for 0, 7 or 28 days) on concentrations of the three most commonly measured electrolytes (sodium, chloride and potassium) and three metabolites of recent interest (lactate, ammonia and urea) in sweat.

## Materials and methods

Procedures were approved by the Ethics Committee of the Faculty of Behavioural and Movement Sciences of the Vrije Universiteit Amsterdam (VCWE-2020-142). The study was conducted in accordance with the guidelines of the revised *Declaration of Helsinki* (2013). Written informed consent was obtained from all participants before participation in the study.

Twelve healthy individuals (6 males, 6 females; characteristics presented as median (range); age: 29 (23–35) years; height: 174 (164–195) cm; weight: 68.6 (57.2–104.6) kg) participated in this study. Participants were instructed to refrain from alcohol 24 h before the experiment, to limit caffeine consumption and to consume plenty of water before starting the experiment. No other restrictions were placed on their diets. All participants were nonsmokers, did not take any prescription medication, had no history of heat-related illnesses, cardiovascular complications and did not have any known issues with thermoregulation. Menstrual cycle phase was not controlled for in the female participants as this is not expected to interfere with the influence of storage temperature and duration on sweat composition.

Participants visited the laboratory once to cycle (Lode Excalibur, Groningen, The Netherlands) for 60 min at 40 W.m^−2^ [29]. Experiments took place in a climate chamber (b-Cat, Tiel, The Netherlands) set to 33°C and 65% RH. To allow for a washout period of the skin [1], 20 min after the onset of exercise six absorbent patches (25 cm^2^) were applied to the posterior torso. During this 20-min period, participants were already sweating profusely. To prevent epidermal contamination, the skin was thoroughly cleaned with alcohol, deionized water and dried with gauze pads before application of the patches. The researcher wore gloves while applying and removing the patches. The absorbent material was only touched with cleaned tweezers. Patches were applied to both scapulae (region 14 as reported by [30]; two in total) and the lower back (regions 16 and 18 as reported by [30]; four in total). After collection, samples were centrifuged (1800 *g* for 5 min at 4°C; Universal 32 R, Hettich Benelux, Geldermalsen, The Netherlands). Sweat from the six samples was mixed, centrifuged again and divided equally over six vials using a pipette. To circumvent the occurrence of epidermal contamination, it was verified that the sweat was clear without any supernatant on top after centrifuging. Sweat composition of one sample was determined immediately in a biochemistry laboratory and was used as control in evaluating the influence of storage temperature and duration. Two samples were stored at room temperature (approximately 25°C, 42% RH) at the biochemistry laboratory for 7 and 28 days respectively. The remaining samples were frozen at −20°C for 1 h, 7 or 28 days before analysis. Freezing samples at −20°C was chosen because the standard procedure for short-term (< 3 months) storage of bodily fluids in a biochemistry laboratory is −20°C and typically storage at this temperature is required for the intended chemical analyses [31]. On the other hand, in practice, it is not always a possibility to freeze samples. Therefore, samples are sometimes stored at room temperature until chemical analysis. The selected storage durations (0, 7, 28 days) were chosen to allow for comparison with previous research [11]. In addition, storage for 7 days is a realistic time span for field work and 28 days was selected to determine the impact of a significantly longer storage duration.

Upon arrival to the laboratory, participants provided a urine sample to confirm hydration status. Urine specific gravity (USG) was measured with a handheld refractometer (PAL-S, Atago, Bellevue, USA) (USG ≤ 1.020) [32]. For sweat sampling, the absorbent material (Cutisoft, BSN Medical, Almere, The Netherlands) was covered with an impermeable layer (Parafilm-M, Bemis, Saint Louis, USA) and was attached to the skin by a porous adhesive (Fixomull stretch, BSN Medical, Almere, The Netherlands). Prior to application, the skin of the back was rinsed with alcohol and deionized water and dried with gauze pads. Patches were carefully applied whilst the researcher wore gloves and only touched the absorbent with tweezers. Sweat samples were collected for ~25 min to collect enough sweat for chemical analysis. To prevent saturation, patches were visually inspected and removed earlier if necessary. After removing the six absorbents from the skin, they were placed in clean airtight tubes (Salivette, Sarstedt, Nümbrecht, Germany). To extract the sweat, the six tubes were centrifuged (1800 *g* for 5 min at 4°C). Sweat was collected in one tube, centrifuged once more (1800 *g* for 5 min at 4°C) and subsequently equally divided over smaller vials (Cryogenic vials, Greiner Bio-One, Alphen aan den Rijn, The Netherlands) using a pipette (FinnPipette^TM^, Thermo Fisher Scientific, Waltham, USA).

One of the primary concerns regarding sweat sample storage is evaporation of the water portion of the sample which may cause increases in measured concentrations [13]. To prevent evaporation, it is recommended to seal the samples during storage using the impermeable fabric Parafilm-M [9,13], which was done accordingly. Before carrying out the analyses, sweat samples were thawed using a roller shaker for 15 min. Concentrations of sodium, chloride, potassium, ammonia, lactate and urea were determined on Cobas analyzers (Roche Diagnostics, Almere, The Netherlands) by ion-selective electrodes for electrolytes, by an enzymatic method for ammonia and lactate, and by a kinetic method for urea.

Statistical analyses were performed using IBM SPSS Statistics 26.0. Effects were considered significant if P < 0.05. Data are presented as means and standard deviations (SD). The Shapiro-Wilk test was used to check if the data was normally distributed. A repeated-measures ANOVA was used to assess whether sweat composition (sodium, chloride, potassium, ammonia, lactate and urea concentrations) differed due to storage condition (5 levels: 25°C for 7 days, 25°C for 28 days, −20°C for 0 days, −20°C for 7 days or −20°C for 28 days) compared to control (immediate analysis). Violations of sphericity were corrected using the Greenhouse-Geisser adjustment. Where main effects occurred, Bonferroni corrected pairwise post-hoc comparisons were made. As an expression of variability, intra-assay coefficients of variation (CV) were also calculated to assess differences between the different storage conditions and control.

## Results

**Figure 1.**
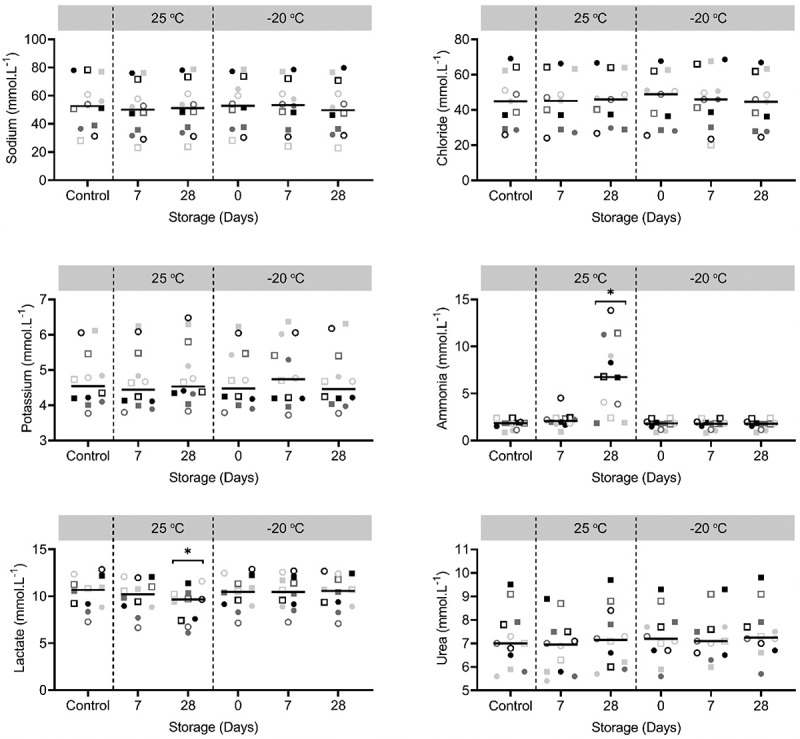
Concentrations of sweat sodium, chloride, potassium, ammonia, lactate and urea after several storage conditions (25°C for 7, 28 days or −20°C for 0, 7 or 28 days) compared to control (i.e., immediate analysis). Each symbol in a specific color represents one individual. Black horizontal bars represent means (*n* = 12). * Indicate significant differences (P < 0.05) compared to control

All participants completed the protocol and sweat was successfully collected from the posterior torso. Individual concentrations of sweat sodium, chloride, potassium, ammonia, lactate and urea are shown in Figure 1. Compared to control, there were no significant main effects of storage condition on sweat sodium (P = 0.059), chloride (P = 0.077), potassium (P = 0.134) or urea (P = 0.124, Figure 1). There were, however, significant main effects of storage condition on sweat ammonia (P = 0.001) and lactate (P = 0.006) concentrations (Figure 1). Post-hoc testing revealed that sweat ammonia concentration was significantly higher compared to control after storage at 25°C for 28 days (5.1 ± 3.9 mmol.L^−1^, P = 0.017, Figure 1). Post-hoc testing likewise revealed that sweat lactate concentrations was significantly lower compared to control after storage at 25°C for 28 days (−1.8 ± 1.8 mmol.L^−1^, P = 0.007, Figure 1).

## Discussion

This is, to our knowledge, the first study that investigated the effect of five sweat sample storage conditions (25°C for 7, 28 days or −20°C for 0, 7 or 28 days) on six biomarkers in sweat: sodium, chloride, potassium, ammonia, lactate and urea. The main findings were that the storage temperature and duration did not affect sodium, chloride, potassium and urea concentrations. However, sweat samples should preferably not be stored for longer than 7 days at 25°C when aiming to determine sweat lactate and ammonia concentrations. When samples were frozen at −20°C, the storage duration could be extended to 28 days for these two components.

Following storage of sweat samples for 28 days at 25°C, ammonia concentrations were higher and lactate concentrations were lower compared to control (Figure 1). Eccrine sweat is sterile and odorless upon secretion but after contamination with components on the skin, bacterial action could occur [33]. The 28 days at 25°C storage condition yield beneficial circumstances for bacterial action, since sweat itself could provide the moisture that is required for bacterial proliferation [34]. Secondly, 25°C is potentially warm enough for proliferation [34,35]. Since ammonia is a waste formed through bacteria, this could explain the high concentrations following storage for 28 days at 25°C. It is unclear why the considerably higher concentrations in sweat ammonia were not observed after 7 days of storage at 25°C. The lag phase (i.e., period of no cell division) of bacterial growth typically takes hours but could also last days [34]. During the lag phase, the bacteria cells are not dormant but instead undergo a period of intense metabolic activity to allow for the subsequent exponential growth phase [34]. It could be that the lag phase lasted more than 7 days and lactate is used as energy source for the increased metabolic activity during that period. This would explain the concomitant lower lactate concentration that was observed in this condition as well. It should be noted that there is considerable inter-individual variability in the effect of storage condition on sweat ammonia concentrations. The bacteria that potentially caused the elevated ammonia concentrations may not be present to the same extent on the skin of every individual, causing large between-individual differences.

To date, the smallest worthwhile or physiological meaningful change in sweat composition has not been pre-determined. However, an acceptable level of agreement seems to be less variability due to storage than previously published values for intra-individual variability. We thus want to know whether the variability of the methods used (i.e., within the 5 levels of storage condition which is called intra-assay variability) is less than previously reported variability within one participant (i.e., intra-individual variability). For sodium, chloride and potassium, previously reported intra-individual variability was ~5–20% [9,36–38]. In the present study, variability associated with the different storage conditions, expressed as CV, ranged from 1.4–4.0% for sodium, 1.5–4.2% for chloride and 0.7–2.5% for potassium (Table 1). These values easily fall within the reported intra-individual variability of ~5–20%. For sweat ammonia, lactate and urea, these intra-variability ranges are not reported yet. Variability for these metabolites was 1.0–50.2%, 0.6–12.1% and 2.2–2.6%, respectively (Table 1). Variability for lactate and urea is comparable to the electrolytes (sodium, chloride, potassium), suggesting that the storage conditions elicited produced acceptable levels of agreement. For ammonia, one storage condition (28 days at 25°C) caused considerably more variability (50.2%) than previously reported intra-individual variability for electrolytes (~5-20%). Storing sweat for 7 days at 25°C also seems to cause more variation in sweat lactate concentrations (12.1%) than freezing (0 days: 1.0%, 7 days: 1.5% and 28 days: 1.0%).

**Table 1.** Mean (SD) differences and intra-assay coefficients of variation (CV) in sweat sodium, chloride, potassium, ammonia, lactate and urea concentrations after storage at 25°C for 7 days, 25°C for 28 days, −20°C for 0 days, −20°C for 7 days or −20°C for 28 days compared to immediate analysis (i.e., control) (*n* = 12)

		25°C				−20°C					
	Control (mmol.L^−1^)	7 days		28 days		0 days		7 days		28 days	
		Mean difference (mmol.L^−1^)	CV (%)	Mean difference (mmol.L^−1^)	CV (%)	Mean difference (mmol.L^−1^)	CV (%)	Mean difference (mmol.L^−1^)	CV (%)	Mean difference (mmol.L^−1^)	CV (%)
Sodium	53.4 (17.1)	−3.3 (1.6)	3.7	−1.6 (1.9)	2.2	0.2 (2.9)	1.4	0.0 (5.4)	3.5	−2.4 (2.8)	4.0
Chloride	45.4 (14.4)	−0.7 (1.3)	1.5	−0.1 (1.6)	1.6	0.0 (2.0)	4.2	4.0 (7.1)	4.3	−1.2 (1.2)	1.7
Potassium	4.7 (0.8)	0.0 (0.1)	0.9	0.1 (0.1)	1.6	0.0 (0.2)	1.1	0.2 (0.5)	2.5	0.0 (0.1)	0.7
Ammonia	1.7 (0.5)	0.5 (0.7)	11.1	5.1 (3.9)	50.2	0.0 (0.0)	1.0	0.0 (0.0)	1.5	0.0 (0.0)	1.0
Lactate	10.3 (1.7)	−0.3 (0.3)	1.9	−1.8 (1.8)	12.1	0.0 (0.2)	0.6	0.1 (0.3)	0.9	0.0 (0.2)	0.9
Urea	7.2 (1.2)	−0.3 (0.3)	2.5	0.0 (0.7)	2.8	0.1 (0.6)	2.2	0.1 (0.6)	2.6	0.3 (0.5)	2.5

Our results are in conflict with previous research on sweat sample storage conditions. We showed no significant differences from the control sample (i.e., immediate sweat analysis) for any of the electrolytes (sodium, chloride, potassium) due to the elicited storage conditions (25°C for 7, 28 days or −20°C for 0, 7 or 28 days). In contrast, Dziedzic et al. [12] reported a ≤ 14% increase in sweat sodium concentration when analyzed immediately after sampling compared to refrigeration at 7°C for 7 days. In the present study, by far the largest mean difference in sweat sodium concentrations was found after 7 days of storage at 25°C: −3.3 ± 1.6 mmol.L^−1^ which corresponds to −7.1 ± 4.5%. This difference is considerably smaller and was not statistical significant. Another study reported a 21–66% increase in simulated sweat chloride concentration when stored for 5 days at 21–23°C compared to immediate analysis [13]. These conditions are comparable to the 7 days at 25 °C condition utilized in the present study, yet we observed minimal changes in sweat chloride concentrations (−0.7 ± 1.3 mmol.L^−1^; Table 1). Our results are in better agreement with findings by Baker et al. [11], who concluded that sodium, chloride and potassium concentrations all decreased slightly (sodium: −0.5 ± 5.3 to −2.1 ± 6.2 mmol.L^−1^, chloride: −0.4 ± 8.2 to −1.3 ± 8.5 mmol.L^−1^, potassium: −0.11 ± 0.45 to −0.15 ± 0.45 mmol.L^−1^) but significantly after 7 days of storage at −20°C, 8°C, 23°C and an alteration of 8°C and 23°C [11].

It is unknown what causes the discrepancy in study outcomes. One possible explanation could be the use of different analytical techniques for the determination of sweat composition. In the present study, ion-selective electrodes were used to determine sweat sodium, chloride and potassium concentrations. This is not a direct measure of concentrations but rather determines electrolyte activity [39]. There appears to be an effect of ionic strength on the measured electrolyte activity and freezing and thawing may potentiate this process, and thus, the ion-selective electrode outcomes. Using ion-selective electrodes, freezing and thawing indeed accounted for a ~7% concentration difference [12]. Previous studies investigating the effect of storage condition on sweat composition all frozen and thawed each sweat sample at most once [11–13], as was utilized in the current study. Furthermore, similar intra-instrument reliability for ion-selective electrodes and ion-chromatography was found [40]. And a high correlation for sweat sodium determined by conductivity measurements and flame photometry techniques was observed [41]. It is, however, not known how the ion-selective electrodes and conductance relate, other than Dziedzic et al. [12] reporting ~20% higher sweat sodium concentrations with conductivity and flame photometry compared to ion-selective electrodes. The discrepancy between studies could therefore (partly) be caused by the different analytical techniques utilized. Secondly, a primary concern regarding sweat sample storage is evaporation which may cause increases in measured concentrations. To prevent evaporation, we sealed the samples during storage [9,13]. The studies mentioned above [11–13] all sealed their vials as well. We therefore do not think evaporation of sweat from the vials helps explaining the discrepancy between studies but we do advocate the use of this technique for future studies to ensure sweat cannot evaporate during storage.

When sweat testing is conducted in the field, sweat samples cannot always be analyzed immediately due to time and logistical constraints. The findings of the present study highlight the need for researchers and practitioners to report detail on their sweat sample storage conditions. The findings of the present study emphasize and contribute to the development of standardized sweat storage conditions to improve reliability of sweat composition data. Our data suggests that when analyzing sweat sodium, chloride, potassium and urea concentrations, samples can be stored at either 7 or 28 days at −20 or 25 °C. Lactate and ammonia should not be stored for longer than 7 days at 25°C. When samples are frozen at −20°C, the storage duration could be extended to 28 days for these two components. It could be that the ~7-10% differences from baseline (Table 1) may be considered important for research focused on relatively small differences in sweat composition. Research should take the accuracy of the analysis, intra-individual variability [9,36–38] and the effects of freezing and thawing into account [12].

A limitation to the present study could be that we based the number of participants (n=12) on the sample size that is typically used in sweat composition experiments in sports science or laboratory research, rather than on a power calculation. In sports science, electrolyte losses are quantified and subsequently an appropriate personalized replacement strategy is selected on an individual level [4,9]. In laboratory research into sweat composition commonly ~5-15 participants are included [26,27,42–44]. The rationale behind this logic is that if the differences cannot be clearly detected in a similar group of participants, it may not be important in practical use.

To conclude, storage temperature and duration did not affect sodium, chloride, potassium and urea concentrations. However, sweat samples should preferably not be stored for longer than 7 days at 25°C when aiming to determine sweat lactate and ammonia concentrations. When samples are frozen at −20°C, the storage duration could be extended to 28 days for these two components.
